# Principlism and Personalism. Comparing Two Ethical Models Applied Clinically in Neonates Undergoing Extracorporeal Membrane Oxygenation Support

**DOI:** 10.3389/fped.2019.00312

**Published:** 2019-07-30

**Authors:** Matteo Di Nardo, Anna Dalle Ore, Giuseppina Testa, Gail Annich, Edoardo Piervincenzi, Giorgio Zampini, Gabriella Bottari, Corrado Cecchetti, Antonio Amodeo, Roberto Lorusso, Lorenzo Del Sorbo, Roxanne Kirsch

**Affiliations:** ^1^PICU, Children's Hospital Bambino Gesù, Rome, Italy; ^2^Clinical Bioethics, Children's Hospital Bambino Gesù, Rome, Italy; ^3^PCICU, Children's Hospital Bambino Gesù, Rome, Italy; ^4^Department of Critical Care, The Hospital for Sick Children, Toronto, ON, Canada; ^5^Interdepartmental Division of Critical Care Medicine, University of Toronto, Toronto, ON, Canada; ^6^Mechanical Assist Device and ECMO Unit, Children's Hospital Bambino Gesù, Rome, Italy; ^7^Department of Adult Cardiac Surgery, Maastricht University Medical Centre, Maastricht, Netherlands; ^8^MSICU, Toronto General Hospital, Toronto, ON, Canada; ^9^Department of Bioethics, The Hospital for Sick Children, Toronto, ON, Canada

**Keywords:** bioethic, ECMO—extracorporeal membrane oxygenation, neonates, principlism and code of ethics, personalism

## Abstract

Extracorporeal membrane oxygenation (ECMO) is a technology used to temporarily assist critically ill patients with acute and reversible life-threatening cardiac and/or respiratory failure. This technology can often be lifesaving but is also associated with several complications that may contribute to reduced survival. Currently, neonates supported with ECMO are complex and bear an increased risk of mortality. This means that clinicians must be particularly prepared not only to deal with complex clinical scenarios, but also ethical issues associated with ECMO. In particular, clinicians should be trained to handle unsuccessful ECMO runs with attention to high quality end of life care. Within this manuscript we will compare and contrast the application of two ethical frameworks, used in the authors' institutions (Toronto and Rome). This is intended to enhance a broader understanding of cultural differences in applied ethics which is useful to the clinician in an increasingly multicultural and diverse patient mix.

## Introduction

Extracorporeal Membrane Oxygenation can be used to electively stabilize neonates from ongoing deterioration because of respiratory and/or cardiac failure or to urgently rescue them in case of cardiac arrest (1–3). ECMO can be established: as a bridge to recovery (in case of reversible disease); as a bridge to a bridge (transition to a ventricular assist device), as a bridge to organ transplantation (rare in neonates); or as a bridge to decision (providing time to recovery, time for diagnosis or time to evaluate candidacy for transplantation, or for a longer term mechanical circulatory support) (4). This rapid expansion of clinical indications (5, 6) has outpaced empirical outcome data; challenging bedside clinical decisions as previously contraindicated disease states (immunocompromised patients, recent surgery, or trauma) are now able to be supported with ECMO (7–10). This emphasizes the need to address important questions such as how decisions to offer ECMO are made as well as how and when to discontinue ECMO when unsuccessful (11–14). In general clinical practice, withholding ECMO (15), even contrary to the wishes of the family or of the legal guardians, is widely perceived as justified, while withdrawal in the face of unsuccessful therapy is often perceived differently by the family, as it requires the action of stopping a currently applied therapy (15, 16). Canadian and American bioethics grants no ethical distinction between withholding and withdrawing therapies. As a therapy, ECMO can therefore be withheld or withdrawn under the same ethical justifications as any pharmacological or technological therapy, such as mechanical ventilation or renal replacement therapy (16–18).

Comprehension of the ethical underpinnings guiding the use of medical technologies, and specifically how this relates to ECMO support in the neonatal period will help clinicians in managing decisions about neonatal ECMO. As medical technology continues to be utilized globally, understanding differences in ethical practices can help to inform and enhance the practical application of this complex technology and the evolution of guidelines for its use. We start by outlining the ethical framework of Principlism (19) and how it is applied in clinical decision making in neonatal ECMO. Then, we introduce the less globally known Personalist bioethics (20, 21) framework. We will outline pitfalls and limitations in both and demonstrate how each affects practical use of this complex resource.

## A Brief Review of Principlist Bioethics in Clinical Application

**Figure 1:**
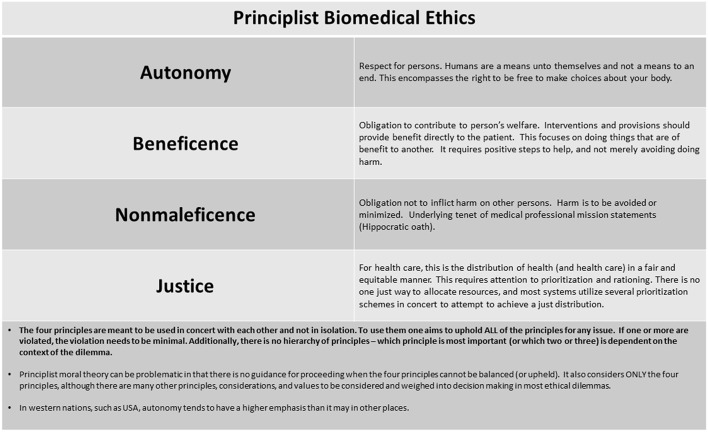
Principlist biomedical ethics.

The health care team in a Principlist bioethics system seeks to balance four principles of autonomy, beneficence, non-maleficence, and justice (19) when making treatment decisions (Figure 1). Additionally, an overarching additional concept of best interests is applied to patients incapable of participating in decision making, due to age, illness, or cognitive capabilities. Ideally, the therapy is desired by the patients, it provides benefit to them, avoids unjustified harms, the benefits outweigh harms, and medical resources are allocated in a fair manner and most often with maximization of benefit. Before initiating ECMO, wherever possible, a detailed and informed consent is undertaken with the parents or legal guardians, communicating risk, benefits, alternatives and including information on the potential for ECMO to be unsuccessful in achieving the intended goal.

## The Principle of Autonomy

Central to the ethical provision of any medical intervention is the principle of autonomy, which upholds respect for persons. This provides the basis for informed consent (19). In a practical sense, autonomy refers to the individual's rational capacity for self-determination (19). Each patient can express his preferences about therapies and his reasoning in accepting or refusing them. To uphold this principle, the health care team needs to respect the wishes of patient (presuming they have capacity) regarding medical interventions.

The sick neonate is considered a “vulnerable” patient because is unable to express his will. However, this limits only in part the application of this ethical principle because the guarantors of this principle are the parents supported by the best knowledge of the physicians. The physician “must” wisely inform the parents and act consequently taking into account all the other bioethical principles (beneficence/non maleficence and justice). Only a strong and sincere dialogue between the physicians and parents will allow to understand the real needs of the neonate.

In many countries, such as Canada, United States, United Kingdom, and others, it is both ethically and legally permissible to forgo or request discontinuation of life-sustaining therapy, even if the intervention is life prolonging or beneficial (4, 5). Otherwise said, they can choose not to act for their own benefit. This practice is supported by ethical concepts (e.g., Kan's moral theory, etc.) beyond and in addition to the framework of Principlism, although our discussion will focus only on Principlism within the scope of this article (5).

## The Principle of Beneficence

This principle obligates the health care team to contribute to the person's welfare with any interventions conferring benefit directly to the patient (19). Benefit for the patient, however, may reach beyond medical outcome measures. The values, beliefs and culture of the patient may modify how benefit is perceived. For example, a patient may decide that a specific therapy is not beneficial because the resultant residual morbidity is untenable and will not allow a “good” life. This can be understood more readily in the patient with capacity who can express how these values influence their preferences about medical therapies.

Benefit for the patient without capacity, however, is generally not only regarded under a principle of beneficence but under an overarching concept of best interests. This is applied to any incapacitated patient, where others have to make choices that affect him on his behalf, including a child or neonate.

Best interests become more difficult to decipher, therefore, when encompassing both medical outcome and values or preferences expressed by the surrogate decision maker on behalf of the patient. This is particularly true for the child or neonate, who is incapable of having yet expressed any wishes or values. The child or neonate, because they exist within their family's values, beliefs and culture, is most likely to choose in accordance with their family and will leave a medical experience to exist within their family context. Therefore, family surrogates are generally the best choice to represent a view of the child's best interest. Additionally, the pediatric clinician is charged with protection of the child and may question the family's view as truly being in the child's best interest. In this instance they can bring into question the decisional authority of the family. If both parties have moral grounding for their judgment of best interest, however, the determination becomes even more complicated (4–6, 22, 23).

## The Principle of Non-maleficence

The principle of non-maleficence renders an obligation not to inflict harm on any person (19). Should it be impossible to avoid harm, it should be minimized, and therefore benefits of an intervention should outweigh the risks and the intendent suffering of the intervention. For example, during a neonatal ECMO run, the risks of neurological disabilities, where known are disclosed in advance before the ECMO deployment. These potential comorbidities are acknowledged by the family, especially when the neonates have other risk factors contributing to worsen their neurological outcome (prematurity, low birth weight, coagulopathy, etc.). If the probability of neurological disability is high using ECMO, or the probability of poor outcome (death or severely reduced quality of life) for the patient in general is high, the procedure may not be undertaken, as it would provide risk of harm without any benefit of longer term good outcome (4, 6).

## The Principle of Justice

The principle of justice as it relates to health care considers the obligation to fairly and equitably distribute health and health care. This requires prioritization and rationing of competing claims (19). In health care this might be subdivided into categories of: fair distribution of scarce resources (distributive justice), respect for people's rights (rights based justice) and respect for laws (legal justice) (24). Health care providers are challenged to use resources wisely and to grant equality and equity to all sick people. The right to be treated equally and with equity can be found in many constitutions, but in the actual practice, a number of different factors may influence the access to treatment (e.g., age, place of residence, social status, ethnic background, culture, sexual preferences, disability, legal capacity, hospital budgets, insurance cover, and prognosis). The principle of justice regulates these aspects in order to avoid any form of discrimination and to provide to all the people the same respect. The sick neonate is often considered a “vulnerable person” because is still not actively part of society and is dependent on parents to survive (incapable of self-determination). However, when dealing with ECMO, this issue is solved using the dedicated “clinical criteria” which grant a fair access to this life support technique even in the less “wealthy” countries.

Good resource allocation must ensure processes that distribute or deny therapies in a fair fashion. There is no single agreed upon prioritization scheme with continued debate upon the best ways to make allocations. Generally, a combination of prioritization schema are required to attempt to achieve a just distribution. Distributing based only on quality-adjusted life years (QALY) saved, or cost-effectiveness allows for comparisons, but still requires subjective judgments about quality of life, and often fails to account for unmeasurable benefits (or harms) to the society or system (4, 25–29).

## Considerations on Principlism

**Figure 2:**
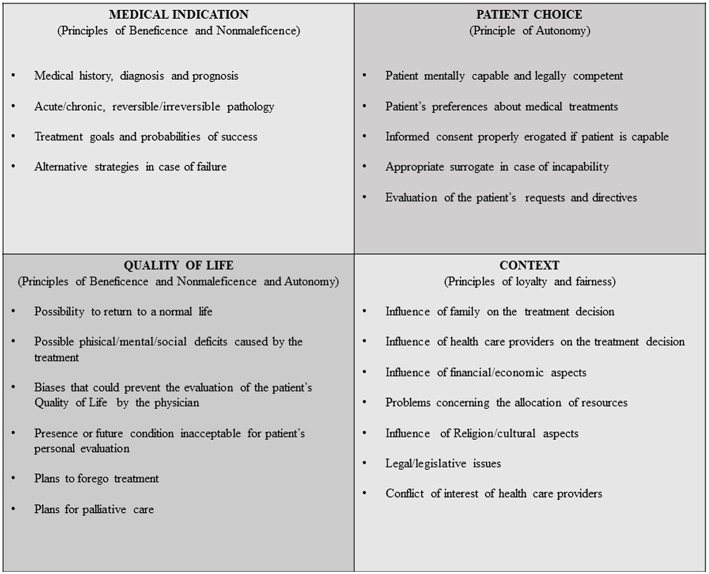
Facilitating ethical and medical practice—the 4 box method.

The term principle has wide ranging significance. In bioethics, this term is generally used in reference to the four principles of Beauchamp and Childress, previously addressed (19, 24, 30, 31). In any given ethical deliberation, the principles are meant to be simultaneously upheld, but if one or more principle should be violated, such violation should be minimal and mitigated where possible. However, with no hierarchical structure between the principles, they are left subject to a certain relativism that can be problematic when applied to the complex scenarios of clinical medicine and biotechnology. To overcome these limits, Jonsen et al. (32) proposed the four Box-Method to help clinicians to organize the ethical reasoning in medical indications, patient preferences, quality of life, and contextual features. These four topics provide a pattern for collecting, sorting, and ordering the facts of a clinical ethical problem. Each topic can be filled with the actual facts of the clinical case that are relevant to the identification of the ethical problems. The contents of all four topics viewed together form a comprehensive picture of the ethical dimensions of the case (Figure 2).

When managing complex decisions, the health care team determines which interventions might be offered to alter the course of the medical illness, considering potential survival, comorbidities, and overall quality of life outcomes so as to best inform the patient (or their surrogate decision makers) of the recommended intervention and alternate options (27, 28). Additionally, they try to understand patient or family values and preferences about what constitutes a “good life” (or an acceptable quality of life). In a deliberative process the clinician and patient (or family as surrogate decision maker) then ideally arrive together at an agreement for how to proceed with therapies (29). Disagreements may occur, and a relatively wide latitude is given to many choices of the parent or surrogate decision maker particularly when there is not a high probability of restored health or good outcome. The clinician has the additional task to question the decisional authority of the parent or guardian in instances where they do not feel the best interests of the patient are being upheld. However, disagreements may occur. Should the clinician feel the best interest of the child would be to stop ECMO for reasons of no benefit and induced suffering, while the family holds a stance on the sanctity of life even at a high degree of suffering, an impasse will appear that requires careful mediation and at times, legal determination.

Finally, most health care systems utilizing Principlist ethical justifications do not use it exclusively in any given ethical dilemma, since it considers only the four principles. For a fulsome ethical deliberation, applying the many other principles, moral theories, values and considerations of the issue at hand are required.

## Introduction of Personalist Bioethics

**Figure 3:**
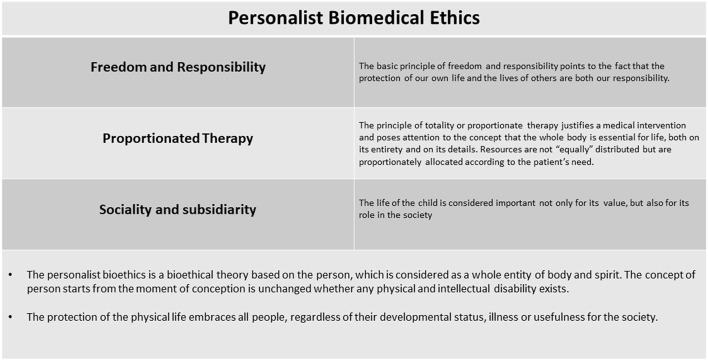
Personalist biomedical ethics.

Personalist bioethics was born in Italy, in a catholic context, to deal with the progression of medicine and the complex challenges it presents. The ontologically grounded Personalism was developed from an anthropological point of view by the Pope Karol Wojtyla and from a bioethical point of view by Sgreccia (20). In the personalist perspective life is considered *sacred* and is at the basis of any bioethical discussion. The person is regarded as an entity of both body and spirit (21, 31). Personhood starts from conception and remains unchanged by physical or intellectual disability. Importantly, Personalist bioethics should not be confused with theories of *individualism* (21), which considers the main constitutive feature of the person to be their capability for individual decision. Personalism is based on the principle that *all* human beings deserve respect (20, 21). Personalist bioethics therefore integrates the concept of the protection of the physical life with other bioethical principles commonly used to manage many current medical challenges (Figure 3).

Personalist bioethics is articulated in three ontologically-based principles: (a) the principle of freedom and responsibility, (b) the principle of proportionate therapy, and (c) the principle of sociality and subsidiarity (8). All three can be considered as corollaries of the main concept of the personalistic bioethics which regards the protection of the physical life. The body as well as the spirit is essential to the person, as it is the first embodiment in which and by means of which, the person is realized and enters into time and space, expresses and manifests himself.

The protection of the physical life embraces all people, regardless of their developmental status, illness or usefulness for the society. The sick neonate is a physical person independently if 1 day he will be able to express his autonomy, thus both the health care team and the family have the duty to protect his life (21, 33). The defense and promotion of life has its limit in death, which is part of life, and health promotion has its limitations in the disease. When disease is incurable you have to take care of the sick person.

## The Principle of Freedom and Responsibility

This principle requires that the protection of our own life and of the life of others are both under our responsibility (20, 21). The concept of responsibility is not detached from the concept of freedom. Freedom means the liberty to take the responsibility for one's own life first and foremost, as well as for the life of others.

In Personalist bioethics, the principle of freedom always requires informed consent (34). Informed consent means full information to the patient, patient's family or to the legal guardians before the intervention, but the responsibility for the final decision to pursue or forgo an intervention is shared between the heath care team and the family. The clinician always asks informed consent before doing an intervention on the patient, respecting the patient's freedom, while the family must always respect the freedom of the doctor to work with responsibility and consciousness. The family *cannot* choose for the child's death when there are opportunities to sustain and protect life (20). This would misuse the concept of freedom because it does not appropriately care for the child's life. In the personalist bioethics life is always sacred and must be respected. Thus, in certain cases, such as when a family refuses a treatment essential to the neonate's life and the physician has deemed the treatment as necessary in good conscience, this principle must govern the procedure for “obligatory care.” However, first and foremost the personalist bioethics seeks a “therapeutic cooperation” (35, 36) between the heath care team and the family to overcome any divergence.

## The Principle of Proportionate Therapy

The principle of totality or proportionate therapy justifies a medical intervention and poses attention to the concept that the whole body is essential for life, both on its entirety and on its details (20). The principle of inviolability of life which has been shown to be primary and fundamental is not disproved but applied when it becomes necessary to intervene in a harmful manner on part of the body in order to save the whole and the very life of the subject. This principle ultimately upholds all the legitimacy of medical and surgical treatment in the Personalist theory. Thus, any intervention on the physical life is justified only if it has a therapeutic purpose to improve the physical life of the patient.

The use of ECMO in neonates is justified, even though invasive and with associated risks, only if it is aimed to allow for cure of a disease state or as a bridge to diagnosis. When ECMO goals are no longer achieved, the intensity of care is reduced. Maintaining ECMO when there is no longer a chance of survival would represent an unjustified “gravamen” (burden) for both child, family, and often the health care team. Personalist bioethics, therefore, suggests a reduction of care. This requires utilizing the concept of proportionate care (20, 21, 31, 35, 36). Here, a treatment must be evaluated within the totality of the person in order to apply or continue it; moreover, there must be a certain proportion between the risks and damages it entails and the benefit it secures. Certain conditions are required to apply this principle: (a) the intervention on a part of the whole body can be performed only in order to save the healthy organism; (b) there is no way or means to correct that condition: (c) there is a good and proportionate chance of success; (d) the patient or the family (legal guardians) have provided consent.

## The Principle of Sociality and Subsidiarity

The principle of sociality implies that all the citizens work toward respecting their own lives and the lives of other as good—not only a personal one but also a social one—and that they engage the social community to promote the life and the health of all, promoting the common good by promoting the good of each individual (20, 21). In terms of social justice, however, the principle obligates the community to guarantee everyone the means of accessing necessary care, even at the cost of sacrifices for the well-to-do. The principle of sociality melds with the principle of subsidiarity, whereby the community must help more where the need is greater (35, 36).

## Considerations on Personalism

The first characteristic of Personalism is that all the principles refer to a well-defined anthropological theory, the defense of the person's physical life, the second is that all the other principles are considered corollaries of this main aspect (21, 35, 36). This anthropological theory is based on the concept of person, regardless of his functions, conscience, race, sex, and stage of development. In this case, the newborn period represents a stage of the physical life, where the individual is already a person; thus, this implies a profound attention and care. This point is very delicate, especially when a neonate is on ECMO and this support is failing to reach its goals. According to the personalist bioethics the first thing that we always have to guarantee is the protection of the physical life but, we must not maintain life at all costs. For a neonate when there is no further chance of survival and the body has not responded to therapies, continuing does not respect the physical person, but rather represents a therapeutic stubbornness, or a desperate search for “vitalism,” which is not accepted in Personalist bioethics. To avoid such a search for “vitalism,” Personalist bioethics considers additionally, the principles of proportionate therapy and of sociality and subsidiarity, to respect the sanctity of life. Only from the integration of these two principles can the sanctity of life be fully respected, especially in neonates who are unable to express their autonomy.

Unfortunately, many conflicts are often unresolvable using these principles (19–21), since there is not a unified moral theory from which these principles are derived (24, 30). By accepting this critique, the personalistic bioethics proposes the “personalistic norm of morality” (20) to order its principles and to work out an integral theory of the dignity of the person to manage fundamental problems in bioethics.

For proponents of Personalist bioethics, additional theories may contribute to a continuing ethical deliberation regarding complex medical scenarios, but the central point of the concept and value of the person that is key to Personalism is generally felt to be missing in other theories.

## Withhold and Withdrawal of Life Support in Principlist and Personalist Bioethics

Neonatal ECMO when not immediately able to provide good outcome or successful discontinuation of ECMO, can lead to internal and external conflicts in determining a time to stop. This aspect introduces the considerations of futility. Futility considerations have long been argued in the medical literature, but remain ill defined (23, 27–29, 37, 38). In the recent era, futility is most often considered when the goals of a medical intervention cannot be or are not achieved (physiologic futility) (37, 38). This moment is then associated to a consideration of withhold or withdrawal of ECMO as appropriate. Most often in the Principlist bioethics, withhold or withdrawal are determined in an intersection of beneficence and non-maleficence, where autonomy dictates respect for the person so as not to continue therapies that are not of benefit to them. If no benefit for the person can be achieved and harms are being accrued, it is deemed appropriate to stop. The best interest of the patient can no more be accomplished because the purpose of ECMO support is lost or the quality of life is significantly reduced without anticipation of benefit.

This can be compared with Personalist bioethics, which would not consider the quality of life of the patient (in present or future) and would continue ECMO while any chance to cure the patient is still present (39, 40). Nonetheless, Personalist bioethics, would agree with stopping ECMO to avoid suffering when there is no longer a chance to cure.

This provides only the briefest discussion of the equivocal and controversial nature of the term futility and the extensive difficulties in understanding and applying this concept (41). Concerns for futility extend well-beyond ECMO therapy and considerations for how to deal with such concerns are multiple and require careful communication and processes for mediation. This is not unique to any institution nor is it solved in practice by any singular bioethical consideration. A more thorough consideration reaches beyond the scope of this manuscript and is well-outlined elsewhere within the literature.

## Decisional Authority in ECMO

**Figure 4:**
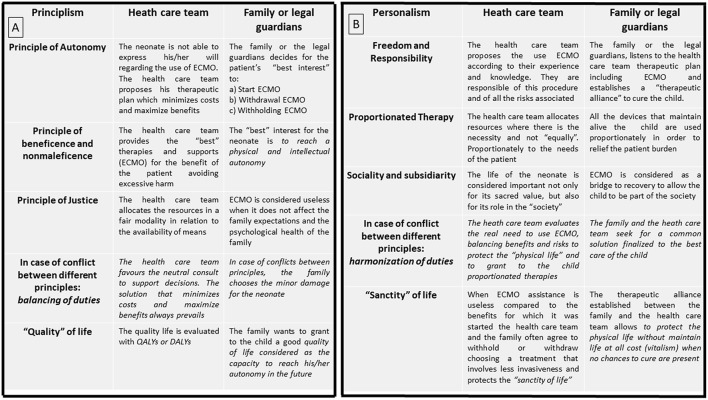
**(A)** Principlist Bioethics method applied at bedside while a neonate is undergoing ECMO. **(B)** Personalist Bioethics method applied at bedside while a neonate is undergoing ECMO.

Principlism and Personalism utilize different principles to manage complex clinical scenarios and parental input (Figure 4). According to Principlism, the autonomy of the patients is respected in part by involving them or their surrogate (parents) in a shared decision (4, 5, 10, 28, 29, 33) to determine how to intervene for the best interest of the patient. A collaborative process that allows both the family and clinicians to reach a common health care decision has been proposed and endorsed by many international medical and nursing societies (41). In Personalist bioethics, a shared decision is also sought, but here, the main goal between the two parties (clinicians and family) is always the respect and the protection of the physical life. Both clinicians and family must protect the patient's dignity with a proportionate care to avoid suffering when there is no longer a chance for life (35).

Divergences or disagreements can come from both health care teams and patients or families (29, 42, 43). Both Personalist and Principlist bioethics concepts support mediation of disputes (2), and both look to maintain a therapeutic alliance, advocating regular communication from the very beginning of the ECMO course.

## Conclusions

Applying ECMO to a patient is a medical act, invading the person and inducing a degree of suffering even though it is done in the interests of saving a life. It is therefore inherently a moral act. Like other therapies, a choice between doing “good things” (beneficence) and avoiding “bad things” (non-maleficence) is inherent to ECMO. In these complex scenarios the contribution of bioethics is fundamental to analyze the ethical underpinnings that support decisions and actions. We have attempted to briefly outline the main tenets of both Principlism and Personalism to understand differences and similarities in how these concepts are applied clinically. Overarching both is a clear support for therapeutic interventions that improve patient health. Key differences lay in the emphasis between quality of life and sanctity of life, although neither finds sanctity of life absolute and to be accomplished at all costs. Personalism considers the protection of life first and foremost qualified after by proportionality. Principlism considers the four principles in concert but without hierarchy, although in practice relies additionally on other ethical theories, frameworks, values and considerations to accomplish the best interest of the patient.

All can agree, regardless of bioethical application, that the neonate has no capability for autonomous expression. In Principlism, intervening for the patient's best interest represents a part of the principle of autonomy. In Personalism, the focus is on life first and foremost and a consideration of autonomy would be inappropriate for the neonate. The personalist system helps both clinicians and parents to protect life as a priority even in the face of escalating comorbidities but allows withhold or withdrawal when there is no further chance for life, in practical, when the goals for which ECMO was started are lacking. Given the complexity of medical intervention, conflict is bound to arise regardless of system. Both frameworks agree that communication, empathy, and support of the family are critical to delivering high quality care. In discussing the similarities and differences in applied ethics of both Principlism and Personalism in our cities of Toronto and Rome, it is hoped a broader understanding of cultural differences and ethical justifications can enhance the care of an increasingly multicultural and diverse patient population.

## Author Contributions

MD conceptualized the project and MD, AD, and GT wrote 50% of the manuscript. RK wrote the remaining 50% of the manuscript and provided oversight to form and structure. AA, EP, GZ, GB, CC, RL, GA, and LD provided revisions to the manuscript.

### Conflict of Interest Statement

The authors declare that the research was conducted in the absence of any commercial or financial relationships that could be construed as a potential conflict of interest.
